# The effect of corporate social responsibility on trustful relationship, supportive communication intention, and brand loyalty of ethnic halal restaurants

**DOI:** 10.3389/fpsyg.2022.1022835

**Published:** 2022-10-13

**Authors:** Davron Toshmirzaev, Young-joo Ahn, Kiattipoom Kiatkawsin, Ian Sutherland, Seweryn Zielinski

**Affiliations:** ^1^The College of Hospitality and Tourism Management, Sejong University, Seoul, South Korea; ^2^Business Communication and Design, Singapore Institute of Technology, Singapore, Singapore

**Keywords:** corporate social responsibility (CSR), trust, supportive communication intention, ethnic restaurant, brand loyalty, halal food

## Abstract

The purpose of this study is to examine the relationships among CSR activities, brand trust, supportive communication intention, and brand loyalty in a halal restaurant franchise. This study attempts to understand the brand loyalty formation among foreigners visiting a halal ethnic restaurant franchise in South Korea. This study can contribute to the body of empirical evidence showing that CSR activities for different stakeholders can increase brand loyalty mediated by trust. Data were collected from an online survey platform, and visitors were recruited in one of the largest halal franchise restaurants in Korea. This study used a convenience sampling technique, and 225 questionnaires were used for final analysis. Structural equation modeling was likewise used in this research. The results reveal that CSR activities include four constructs: for communities, employees, the environment, and customers. These four dimensions reflect voluntary activities for core stakeholders and are positively associated with enhancement of trustful relationships between customers and restaurants. Among four CSR dimensions, CSR activities to employee are the most influential dimension, followed by CSR to community and to environment. However, the insignificant effect of CSR activities to customers on trust is found. Furthermore, trust positively influences supportive communication intention and brand loyalty. The proposed model of this present study can shed light on how to enhance brand loyalty and sustain trustful and emotional value of an ethnic restaurant franchise. The results can provide important implications for planning CSR engagement from a practical and managerial perspective in the restaurant industry.

## Introduction

The market size of the foodservice industry in South Korea is rapidly growing (Foodservice and Franchise Agency, 2021). According to South Korea’s Ministry of Trade, Industry, and Energy (MOTIE), the value of the food service market was reported to be approximately 97.02 billion US dollars, representing 6.9% of the GDP and involving 12.56 million employees in 2018 (MOTIE, 2019). However, the market is very competitive, and approximately 64% of franchise restaurants operate for less than 5 years before closing (Foodservice and Franchise Agency, 2021). The food service industry is changing dynamically to meet the fast changing needs of consumers (Mordor Intelligence, 2022). The population of Muslim residents living in Korea and the number of Muslim tourists temporarily visiting non-Muslim countries are increasing (Ministry of Justice, 2022). As a result, the Muslim population is seeking halal food restaurants and services and restaurants serving halal food (Shokhsanam and Ahn, 2021). Muslim-friendly restaurants not only serve halal foods, but also become a place for gathering with community members and holding events because they are closely located in Muslim communities and organizations (KTO, 2021; Trade Times, 2021).

In the competitive restaurant industry, understanding the process of restaurant brand loyalty is a key to maximizing restaurant profits, reducing marketing costs, and building long-term relationships with customers (Kim and Kim, 2019; Kim and Stepchenkova, 2020, 2021). In this regard, the CSR implementation of restaurants is linked to positive outcomes, such as long-term profitability (Inoue and Lee, 2011; Franco et al., 2020). Customers desire to consume brands that match their personal value and show high intention to support CSR activities of companies (Kim, 2017). CSR activities can reflect the core values of companies and enhance the formation of brand loyalty among customers (Ahn et al., 2021). Previous research has highlighted the role of CSR activities and the consequences of CSR engagement (Bhati and Verma, 2020; Ahn et al., 2021). CSR engagement is linked to customer loyalty and improved financial performance (Inoue and Lee, 2011).

Stakeholder theory (Freeman, 2010) has been applied for research on CSR as an important theoretical framework on CSR. Previous research on CSR initiatives in the restaurant, hospitality, and tourism industries have used stakeholder theory (Choi and Parsa, 2006; Inoue and Lee, 2011; Wong and Gao, 2014; Shim et al., 2021). Involvement of CSR programs and various promotions for core stakeholders (KTO, 2021; Trade Times, 2021) are associated with the personal traits and leadership of restaurant owners (Chen et al., 2021). Previous research has indicated the significance of understanding customer perception of CSR practices for stakeholders at restaurant for building a customer relationship and increasing brand loyalty (Dutta et al., 2008; Chatzoglou et al., 2017). The framework of social exchange theory proposed by the seminar work (Blau, 1964) states that trust can be used to improve the understanding of customer perceptions of CSR initiatives as an outcome of the norm of reciprocity when building customer relationships. Previous research has examined the formation of trust through CSR activities (Kim and Kim, 2019; Kim and Stepchenkova, 2020, 2021). Moreover, trust is an important mediator that enables us to understand customer experience and their positive emotional status and behaviors (Uzir et al., 2021). However, there is a lack of empirical evidence, customer perception of key CSR activities, and positive behavior intentions in various restaurant contexts in the hospitality and tourism fields (Bowden, 2009). Moreover, empirical research on CSR dimensions for stakeholders and the formation of restaurant brand loyalty and supportive communication mediated by trust have rarely been observed in different research settings in the hospitality and tourism sectors.

Therefore, the purpose of this study is to examine the relationships among CSR activities, brand trust, supportive communication intention, and brand loyalty in a halal restaurant franchise. This study attempts to understand the brand loyalty formation among foreigners visiting a halal ethnic restaurant franchise in South Korea. This study can contribute to the body of empirical evidence showing that CSR activities for different stakeholders can increase brand loyalty mediated by trust. Furthermore, this study attempts to demonstrate that perceptions of CSR activities can facilitate co-creative behaviors (Bhati and Verma, 2020) among customers and increase their supportive communication intention regarding restaurant CSR programs. This study provides useful information from theoretical and practical perspectives.

## Literature review

### CSR activities

Corporate Social Responsibility refers to initiatives “that appear to further some social good, beyond the interests of the firm and that which is required by law (McWilliams and Siegel, 2001, p. 117).” Companies build strategies to generate maximal profit as well as to integrate social values and social responsibilities into their strategies (Abd-El-Salam, 2020). CSR has been identified as an essential element that embeds companies in society and means that they cannot be considered separately (McWilliams and Siegel, 2001). The concept of CSR has been studied for decades and positive consequences have been identified for companies actively involving in CSR initiatives (Inoue and Lee, 2011; Jung et al., 2018; Huang and Liu, 2020). For example, positive outcomes of CSR activities include sale promotion and improved financial performance (Inoue and Lee, 2011; Jung et al., 2018), building brand loyalty among customers (Liu et al., 2014; Kim et al., 2017; Huang and Liu, 2020; Kim and Stepchenkova, 2020), improvements in brand image and brand reputation (Kim and Kim, 2014; Kim and Ham, 2016), improved internal and communications marketing (Ham and Lee, 2011; Park et al., 2018; Huang and Liu, 2020; Girardin et al., 2021; Kim et al., 2021) and a decrease in switching intention (Kim, 2018).

However, there is no consensus in regard to the measurement of the dimensions of CSR initiatives (Guzzo et al., 2022). Previous research on the positive impact of CSR initiatives on the restaurant industry has suggested different operational definitions and approaches to the measurement of CSR dimensions (Kang et al., 2010; Inoue and Lee, 2011; Rhou and Singal, 2020; Shim et al., 2021). Previous studies used the CSR activity participation level for their measurement (Jung et al., 2018; Huang and Liu, 2020). One of the mainstream models includes the CSR dimensions, namely, the economical, legal, ethical, and philanthropic dimensions developed by Carroll (Carroll, 1979, 1998). Furthermore, based on the stakeholder theory (Freeman, 2010), the dimensions of CSR related to customer issues, employee issues, suppliers, environmental issues, and community issues were examined (Choi and Parsa, 2006; Inoue and Lee, 2011; Wong and Gao, 2014; Rhou and Singal, 2020; Shim et al., 2021).

However, there has been a lack of empirical studies on CSR perceptions among customers and the consequences of customer behavior (Abd-El-Salam, 2020). In this regard, this study focused on voluntary CSR activities for core stakeholders in the context of the restaurant industry. Consumers can become aware of the CSR involvement of restaurants through passive and active sources and communication, such as company website, new media, campaign, announcement, or labels, and be the actor who communicates CSR activities with others (Ham and Lee, 2011; Kim, 2018). Muslim consumers also need to search for information about ethnic halal restaurants in non-Muslim countries because of their religious beliefs (Han et al., 2019).

Corporate social responsibility for the community refers to participation in voluntary activities to gain community support, such as donating money for community development programs, participating in fundraising, and donating scholarships for students(Choi and Parsa, 2006; Inoue and Lee, 2011). CSR for employees refers to actions that improve health and safety issues in the workplace, provide fair benefits and compensation, and improve human resources (e.g., the recruitment of disabled and minority groups; Choi and Parsa, 2006; Inoue and Lee, 2011). CSR for the environment refers to engaging in practices related to environmental protection, such as enforcing recycling and waste reduction, using less plastic or single-use products, installing water saving and energy saving equipment, and promoting environmentally friendly campaigns (Ham and Lee, 2011; Kim and Stepchenkova, 2021). CSR for the customer refers to the provision of services for customers, such as those that increase customers’ health, show concern for customers’ well-being, and provide a better quality product for consumers (Inoue and Lee, 2011).

### CSR and trust

Brand trust is defined as “the willingness of the average consumer to rely on the ability of the brand to perform its stated function” (Chaudhuri and Holbrook, 2001, p.82).” Previous research indicated that consumers perceive benefits and reciprocity as a result of various CSR activities of organizations. As a result, these CSR activities can create trustworthiness and credibility for customers who perceive that the CSR activities of the organization have had positive impacts and increased the shared value (Choi and La, 2013; Huang and Liu, 2020). Previous research indicates that consumers not only purchase products and services, but also desire to support responsible companies (Ahn and Kwon, 2020). Consumers also tend to evaluate product value through the companies’ CSR activities which increases added value to their products (Kim, 2018). Consequently, the impact of CSR perception of core stakeholders on financial performance becomes evident and pronounced (Inoue and Lee, 2011; Kim and Kim, 2014). Moreover, consumers who are aware of the CSR activities of companies are more included to positively perceive and trust a brand’s image, show positive behavioral intentions, and are less likely to have negative emotions toward these companies (Martínez and del Bosque, 2013; Kim and Ham, 2016; Kim, 2018).

Companies engage in various relationship building activities with core stakeholders (Freeman, 2010), which often yield positive consequences on their cognitive processes (Guzzo et al., 2022). Individuals can perceive the CSR activities of companies through the lens of environmentally conscious programs, such as environmental protection awareness campaigns, waste reduction and energy efficiency, and supporting local products (Boğan and Dedeoğlu, 2019). CSR activities are geared toward different social groups (e.g., vulnerable communities, employees, and individuals) by engaging in humanitarian activities (McWilliams and Siegel, 2001), fair labor practices for employees (Choi and Parsa, 2006; Farrington et al., 2017), and nutrition information, hygiene, health products, and menu choices for customers (Choi and Parsa, 2006; Kim and Ham, 2016; Shim et al., 2021).

Previous research revealed a positive association between CSR initiatives and trust in the restaurant context (Kim and Ham, 2016; Huang and Liu, 2020). For example, Kim and Ham (2016) examined the relationships among restaurant menu labeling and brand trust, brand image, and brand loyalty among casual dining restaurant consumers. The results showed that CSR activities (i.e., providing health food and food information on the menu) increased brand loyalty mediated by brand trust. Kim and Lee (2018) explored the effects of CSR communication on trust, skepticism, and organizational advocacy. The results indicated that CSR advertising and messages enhance trust among customers. Recently, Huang and Liu (Huang and Liu, 2020) demonstrated the role of CSR messages in building customer donation intention and brand loyalty as well as the mediating role of trust. The results confirmed that CSR messages positively influence brand trust. Therefore, this study posits the following statements:

*H1–H4*: CSR activities for community, employee, the environment, and customer have a positive effect on customers’ trust.

### Trust and supportive communication intention

Supportive communication intention refers to “individuals’ intention to actively engage in information-seeking behaviors and word-of-mouth (WOM) communication behaviors, with the aim of demonstrating their interest and support for the company” (Kim, 2017, p. 311). In the restaurant industry, CSR communication with relevant stakeholders is important to enhance the shareholder value and improve the financial performance (Kim and Kim, 2019). As some of the primary stakeholders, customers can support and engage in communication through CSR programs (Pérez and del Bosque, 2015; Kim, 2017; Kim and Stepchenkova, 2020, 2021; Raza et al., 2020; Girardin et al., 2021; Mohammed and Al-Swidi, 2021; Shafiee and Tabaeeian, 2021). Previous research demonstrated that consumers who have trustful relationships with the brand are more inclined to share their experience with others (Bahri-Ammari et al., 2016). Consumers are willing to engage in various activities, such as posting reviews, evaluating and suggesting feedback for companies, and providing useful information for other consumers (Abbas et al., 2018; Chen et al., 2021; Mohammed and Al-Swidi, 2021). Yoon and colleagues (Yoon et al., 2016) examined the relationship of CSR to environment management strategy, organizational trust, commitment, and organizational citizenship behavior. The results found a positive effect of organizational trust on organizational citizenship behavior among hotel employees. Abbas and colleagues (Abbas et al., 2018) examined the relationships among CSR perceptions, customer engagement, and positive WOM, feedback intention, and brand loyalty. The results confirmed that CSR enhanced the cocreation value among customers and increased customers’ engagement in sharing positively about the company with others through WOM and giving proactive feedback to support the company. Therefore, this study posits the following statement:

*H5*: Trust has a positive effect on supportive communication intention.

### Trust and brand loyalty

Brand loyalty is a vital asset in the competitive market (Aaker, 1989, 2003). Brand loyalty is a core advantage related to brand loyalty and customers’ repeated purchasing behavior (Aaker, 1991). It differentiates the brand and highlights its irreplaceable features in customers’ minds (Aaker, 2003). Moreover, highly loyal customers purchase particular brand products and services, tend to show high repurchase intention, and tend to share their experiences with others (Oliver, 1997). As a result, companies reduce marketing costs to retain customers and achieve a sustainable level of competitiveness (Aaker, 1991). However, there is little empirical evidence in this area in the restaurant context. Few studies have pointed out that trust positively influences brand loyalty (Bowden, 2009; Choi and La, 2013; Kim and Ham, 2016; Nikbin et al., 2016; Park et al., 2017; Liu et al., 2019).

For example, Bowden (Bowden, 2009) proposed a conceptual model of customer engagement process for a restaurant brand. The study suggested that trust and affective commitment are positively associated with brand loyalty. Similarly, Choi and La (Choi and La, 2013) investigated the mediating role of trust on CSR initiatives and brand loyalty after a service failure occurs. The results revealed that trust is positively linked with brand loyalty. Recently, Han and colleagues (Han et al., 2020) tested the interrelated associations among CSR, brand image, brand reputation, brand attitude, brand trust, and brand loyalty. The results confirmed the positive effect of trust on brand loyalty. Therefore, this study posits:

*H5*: Trust has a positive effect on brand loyalty.

## Materials and methods

### Survey measures

Three survey questionnaire sections were developed to demonstrate the proposed model. The first section consisted of questions about general restaurant characteristics. The second section included questions on CSR activities for stakeholders, trust, supportive communication intention, and brand loyalty. The final section consisted of questions on demographic characteristics. The research team interviewed restaurant managers to identify the CSR activities being undertaken at ethnic restaurants from a casual Turkish Halal restaurant franchise. Previous research has used CSR dimensions for communities, environment, and employees (Chatzoglou et al., 2017), CSR dimensions for customer health, social, and environment (Dutta et al., 2008), and CSR for environment (Kim, 2017). CSR activity items were used after analyzing the interviews with some modifications from the previous literature (Dutta et al., 2008; Chatzoglou et al., 2017; Kim, 2017). This study included four CSR activities for communities, employees, customers, and the environment. After interviewing the managers of the restaurants about CSR activities and conducting a pilot test, a total of 12 items were included in the final survey, with each construct consisting of three measurement items. These survey items were assessed on a five point-Likert scale (1 = “very disagree” to “5 “very agree”). Brand trust included four items obtained from previous literature (Delgado-Ballester, 2004; Royo-Vela and Casamassima, 2011; Han et al., 2015; Song et al., 2019). Brand loyalty obtained from previous research (Kim et al., 2007; Iglesias et al., 2019) consists of four items. Supportive communication intention was measured with three items (Kim, 2017). The final section included demographic characteristics. The items were measured with a five-point Likert-type scale (1 = “strongly disagree to “5 = “strongly agree”).

### Data collection

The online questionnaire was created on SurveyMonkey, and the research purpose was briefly introduced on the first page of the survey questionnaire. The first section of the questionnaire included screening questions and basic ethnic halal restaurant characteristics. The screening criteria for the participants are as follows: those who (1) had visited the ethnic halal restaurant franchise (i.e., restaurant franchise of this case study) in South Korea within the past year and (2) are at least 18 years old. The characteristics of the restaurants included the location, frequency of visit, and companions. The research team conducted a pilot test with approximately 30 customers to revise and reword the questionnaire items and check the flow of the survey questionnaire. Moreover, graduate students and experts in hospitality and tourism and in the pilot test to increase its face and content validity. The research team confirmed the criterion validity based on the previous literature. All comments and feedback were included in the revision of the questionnaire items, thereby improving the flow of the question items.

This study used a convenience sampling technique. It targeted foreign customers who had visited an ethnic halal restaurant franchise. All question items were written in English. Given that this study focused on CSR activities of ethnic halal restaurant franchises, one of the largest ethnic halal restaurant franchises was chosen as a case study in South Korea (i.e., Kervan restaurant franchises). Customers who search halal foods can easily access several communication channels, such as restaurant information websites, company website, news and magazine articles, travel platforms, and social network service platforms. They can perceive CSR activities of restaurants, visions and missions of the founder, and the national recognition and rewards for socially responsible companies in South Korea through offline and online information sources. The web link of the online survey was posted on online Muslim communities, online foreign student associations, and social media posts. The online survey link was distributed to potential participants from May to June 2020, and a total of 254 individuals filled out the survey. After removing incomplete surveys and those from individuals who did not pass the screening questions (i.e., visited a restaurant from that brand within a year), the research team was able to analyze data collected from 225 respondents.

## Results

### Demographic characteristics

Table 1 presents the demographic information for the respondents. The majority of the respondents were male (*n* = 162, 72.0%), and approximately 28.0% were female (*n* = 63). Regarding age, approximately 49.8% of the respondents fell into 25–29 age group (*n* = 112, 49.8%). The majority of the respondents were in their 20s. Few respondents aged 35 and over participated in this study. Regarding the level of education, approximately 66.2% had a Bachelor’s degree (*n* = 149, 66.2%). Moreover, approximately, 28.0% of the respondents had a post-graduate degree (*n* = 63, 28.0%). Approximately 74.7% of the respondents were single (*n* = 168), and 24.0% were married (*n* = 54). Regarding the annual household income, approximately 43.1% of the respondents earned under KRW 20,000,000 (*n* = 97), and 38.2% had an annual household income between KRW 20,000,000 and 40,000,000 (*n* = 86).

**Table 1:** Demographic characteristics.

Variable	Category	n	%
Gender	Male	162	72.0
	Female	63	28.0
Age (*M* = 25)	18–24	88	39.1
	25–29	112	49.8
	30–34	20	8.9
	35–39	3	1.3
	Over 40	2	0.9
Education level status	High school	10	4.4
	Associate	3	1.3
	Bachelor’s	149	66.2
	Post-graduate	63	28.0
Marital status	Single	168	74.7
	Married	54	24.0
	Other	3	1.3
Annual household income	Under KRW20,000,000	97	43.1
	20,000,000 – less than 40,000,000	86	38.2
	40,000,000 – less than 60,000,000	19	8.4
	Over 60,000,000	23	10.2

*US$1 = 1,243 Korean Won (KRW).

### Testing the proposed model constructs and confirmatory factor analysis

A structural equation model (SEM) analysis is a popular multivariate model (Little et al., 2007; Byrne, 2013; Kline, 2015). A SEM analysis has several advantages. First, it tests a conceptual model proposed based on previous literature and can visualize a path diagram between latent constructs. Second, a SEM analysis can be useful for examining causal relationships between constructs in the conceptual model (Byrne, 2013). Third, it can simultaneously estimate a statistical relationship between independent and dependent variables, mediators, and moderators (Byrne, 2013). Finally, a SEM analysis should follow several steps, such as model specification, identification, estimations of regression coefficients, model fit assessment, and model validation to infer the causal relationships in the conceptual model (Kline, 2015).

In this study, the dimensions were tested by two-factor analysis methods such as EFA and CFA, as recommended by previous research (Anderson and Gerbing, 1988). After analyzing the measurement items, the initial latent factors were identified based on the recommended criteria (Hair et al., 2009). First, the EFA results of CSR indicated that Kaiser Meyer Olkin (KMO) was 0.724 and Bartlett’s test is statistically significant (χ^2^ = 293.370, df = 3, value of *p* = 0.000). Factor loadings of CSR dimensions ranged from 0.852 to 0.585. CSR dimensions included a total of 16 items in the surveys and excluded four items owing to overloading and low factor loadings. Second, the EFA results of trust indicated that KMO was 0.790 and Bartlett’s test is statistically significant (χ^2^ = 351.842 df = 6, value of *p* = 0.000). Factor loadings of psychological empowerment ranged from 0.769 to 0.853. Third, the EFA results of supportive communication intention indicated that KMO was 0.724 and Bartlett’s test is statistically significant (χ^2^ = 293.370, df = 3, value of *p* = 0.000). Factor loadings of psychological empowerment ranged from 0.853 to 0.890. Finally, the EFA results of brand loyalty revealed that KMO was 0.787 and Bartlett’s test is statistically significant (χ^2^ = 296.063, *df* = 6, value of *p* = 0.000). Factor loadings of participation ranged from 0.764 to 0.838. The Cronbach’s Alpha ranged from 0.773 to 0.846. The construct of the lowest Cronbach’s Alpha was CSR for customer and the highest one was supportive communication intention. The Cronbach’s Alpha showed the recommended threshold values (Hair et al., 2009).

**Figure 1:**
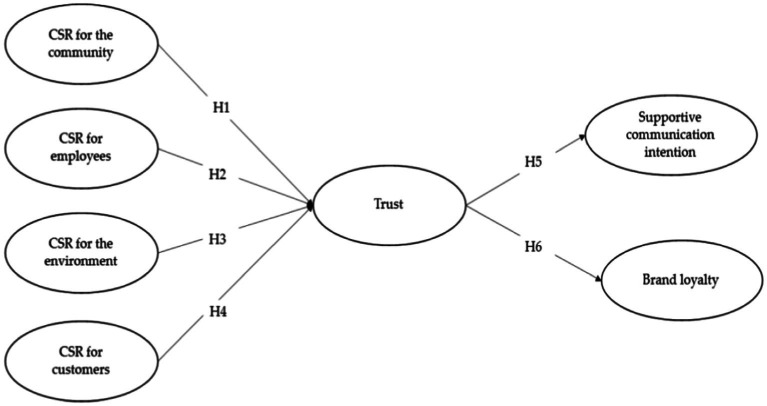
Hypotheses of the proposed model.

Tables 2, 3 demonstrate the validity and reliability of the constructs. Several criteria identified in the CFA results show good model fit. Figure 1 presents the proposed model. SEM was computed to calculate the standardized regression coefficients. The results of this study are calculated by using Stata 16. Factor loadings of CFA are presented in Table 2. The model fits shown in Tables 2, 3 indicate acceptable model fit with the chi-square test and several good fit indices (Bagozzi and Yi, 1988; Byrne, 2013). Composite reliability of the CFA results was from 0.743 to 0.846. The model also showed acceptable levels of internal consistency, composite reliability, and convergent and discriminant validities (Fornell and Larcker, 1981), as shown in Tables 2, 3.

**Table 2:** The measurement items and CFA results.

	Item	Standardized loading
(CSR) Community	The restaurant supports local charities.	0.74
	The __ restaurant supports the community and charitable activities.	0.79
	The________ restaurant supports education.	0.75
(CSR) Employee	The _______ restaurants offers fair treatment of all employees.	0.81
	The _______ restaurant cares employees’ well-being.	0.80
	The _______ restaurant provides employee training for green management.	0.69
(CSR) Customer	The _______ restaurant offers discounts to customers for take-out orders.	0.65
	The _______ restaurant offers a free hot drink (Turkish tea) to customers.	0.62
	The _______ restaurant offers financial support for family meals.	0.82
(CSR) Environment	The _______ restaurant tries to reduce their use of disposable products.	0.72
	The _______ restaurant uses sustainable materials.	0.81
	The _______ restaurant uses energy-efficient equipment.	0.83
Trust	I can rely on the _______ restaurant’s promises.	0.66
	The _______ restaurant guarantees satisfaction.	0.80
	I have confidence in the _______ restaurant.	0.75
	The _______ restaurant is trustworthy.	0.73
Brand loyalty	I intend to continue to visit the _______ restaurant.	0.66
	I think I am very loyal to the _______ restaurant.	0.78
	I would recommend the _______ restaurant to others.	0.64
	I always visit the _______ restaurant, although there are a number of restaurants available.	0.75
Supportive intention	I am willing to discuss the _______ restaurant’s responsible activities with others.	0.82
	I am willing to search for more information on the _______ restaurant’s responsible activities	0.85
	I am willing to pay more attention to the _______ restaurant’s responsible activities.	0.74

**Table 3:** The CFA results and descriptive results of the constructs.

	Items (n)	Mean (Std dev.)	AVE	COM	EMP	CUS	ENV	TRU	LOY	SI
Community (COM)	3	3.76 (0.68)	0.58	0.806^a^	.319^b^	0.251	0.242	0.213	0.185	0.228
Employee (EMP)	3	3.72 (0.71)	0.59	0.102^c^	0.811	0.299	0.341	0.277	0.231	0.369
Customer (CUS)	3	3.76 (0.69)	0.50	0.063	0.089	0.743	0.258	0.167	0.154	0.243
Environment (ENV)	3	3.73 (0.75)	0.62	0.059	0.116	0.067	0.834	0.218	0.196	0.292
Trust (TRU)	4	3.93 (0.60)	0.54	0.045	0.077	0.028	0.048	0.824	0.196	0.203
Loyalty (LOY)	4	3.75 (0.71)	0.50	0.034	0.053	0.024	0.038	0.038	0.801	0.207
Supportive intention (SI)	3	3.89 (0.65)	0.64	0.052	0.136	0.059	0.085	0.041	0.043	0.846

Goodness-of-fit of the model:

χ^2^ (209) = 321.281, p < 0.001 χ2 /df = 1.537; GFI = 0.891; CFI = 0.958; TLI = 0.950; RMSEA = 0.049; SRMR = 0.043.

a is the composite reliability.

b shows the correlation coefficients.

c shows the squared correlations.

### Structural model

**Figure 2:**
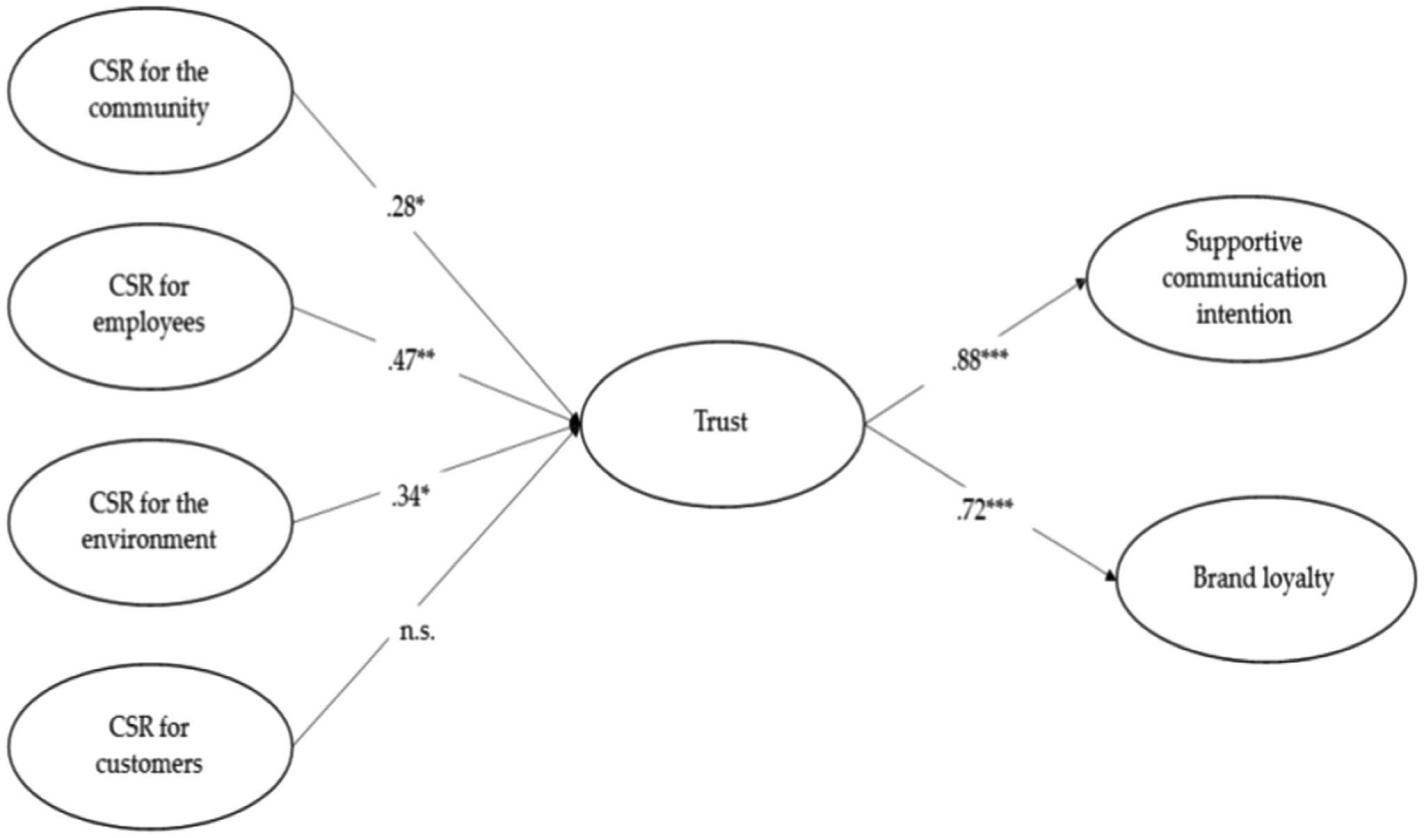
The results of the model.

The results of a SEM are provided in Table 4. The several fit indicates represents satisfactory model fit (χ^2^ = 516.914, *df* = 286, χ^2^/*df* = 1.807, GFI = 0.869, CFI = 0.937, TLI = 0.927, RMSEA = 0.059, SRMR = 0.059; Byrne 2013). The SEM results show that CSR activities for community (β = 0.28, *p* < 0.05) and employee (β = 0.47, *p* < 0.01) are significantly related to brand trust. However, CSR activities for environment (β = 0.34, *p* < 0.05) and customer (β = −0.18, *p* > 0.05) are not significantly related to brand trust. Moreover, CSR activities for employee (β = 0.58, *p* < 0.01) are significantly related to supportive communication intention. However, CSR activities for community (β = −0.10, *p* > 0.05), environment (β = 0.29, *p* > 0.05) and customer (β = −0.05, *p* > 0.05) are not significantly related to brand trust. Trust are positively related to brand loyalty (β = 0.88, *p* < 0.001). Finally, trust is positively related to supportive communication intention (β = 0.72, *p* < 0.001). The results are presented in Figure 2 and hypotheses 1, 2, 3, 5, and 6 are supported. However, a hypothesis 4 is not supported. The R-square for trust is 0.772. The R-square for supportive communication intention is 0.524. Finally, the R-square for brand loyalty is 0.768. The results identified statistically significant indirect effects between CSR activities for community, employees and environments and restaurant brand loyalty and supportive communication intention (Table 5).

**Table 4:** The SEM results of each relationship in the proposed model.

				Coef.	*z*	Hypothesis
H1	COM	→	TRU	0.28^*^	2.19	Supported
H2	EMP	→	TRU	0.47^**^	2.75	Supported
H3	ENV	→	TRU	0.34^*^	1.99	Supported
H4	CUS	→	TRU	−0.18	−1.56	Not supported
H5	TRU	→	LOY	0.88^***^	8.54	Supported
H6	TRU	→	SI	0.72^***^	9.29	Supported

* *p* < 0.05;

** *p* < 0.01;

*** *p* < 0.001.

**Table 5:** Indirect effects of the SEM results.

Indirect paths	Coef.	*z*
COM	→	TRU	→	LOY	0.24^*^	2.14
EMP	→	TRU	→	LOY	0.42^**^	2.81
ENV	→	TRU	→	LOY	0.30^*^	2.44
CUS	→	TRU	→	LOY	−0.16	−1.57
COM	→	TRU	→	SI	0.20^*^	2.17
EMP	→	TRU	→	SI	0.34^**^	2.77
ENV	→	TRU	→	SI	0.25^*^	2.44
CUS	→	TRU	→	SI	−0.13	−1.58

* *p* < 0.05;

** *p* < 0.01.

## Discussion and conclusion

### Theoretical implications

This study examined the relationships among CSR activities, trust, supportive communication intention, and brand loyalty among foreigners visiting a halal ethnic restaurant franchise in a non-Muslim country. A conceptual model was suggested, and empirical evidence provided. The results could have theoretically useful implications. First, the results reveal that CSR activities for core stakeholders in restaurants have positive effects on trust. The multiple dimensions of CSR activities include four constructs, namely, CSR activities for the community, for employees, for the environment, and for customers. These four dimensions reflect voluntary activities by core stakeholders and are positively associated with the enhancement of trustful relationships between customers and restaurants, consistent with previous research (Kim and Ham, 2016; Kim and Lee, 2018; Huang and Liu, 2020). The results found that among the four CSR dimensions, CSR activities for employees are the most influential dimension, followed by CSR for the community and for the environment. The ethnic restaurant franchise may build a positive reputation and continue announcing its CSR activities through restaurant websites, news media, social media platforms, and sponsorships for various community events. Moreover, frontline employees who perceived restaurant CSR activities for employees may provide better customer-oriented services at ethnic restaurants (Phuong and Ahn, 2021).

Second, CSR activities positively influence trust, although an insignificant effect of CSR programs for customers on trust was found. The results may indicate low awareness of CSR for customers and take the CSR activities of restaurant for granted (e.g., free ethnic tea or a discount for the vulnerable and customers). Previous research has examined CSR activities for customers by focusing on customer health concerns, ingredient information, and healthy menus (Chatzoglou et al., 2017). Small- and medium-sized companies (SMEs) in the restaurant industry implement different CSR practices because they have unique tangible and intangible resources and capabilities (Ahn, 2017). Further research may be needed to clarify the perception of CSR activities by customers and other relevant stakeholders. CSR activities based on stakeholder theory (Freeman, 2010) highlight the importance of internal marketing practices for employees and external marketing practices for communities. The results indicated that consumers who build trustful relationships and a level of reciprocity through the CSR activities of restaurants tend to have increased affective brand value and favorable emotions toward the restaurant brand.

Third, trust influences supportive communication intention. Previous research has explained that consumers who trust a particular brand tend to enhance customer experience, meet customer expectation (Uzir et al., 2021), and share their knowledge and experiences with others online and offline (Bahri-Ammari et al., 2016). This study indicated that customers with high-level trust perception are willing to support the CSR activities of a halal ethnic restaurant franchise and share these CSR activities with others. This study focuses on supportive communication intention on restaurant CSR activities for the main stakeholders. Consistent with previous research (Kim, 2017), building trust with customers can facilitate co-creation behavior among customers who communicate their restaurant experiences and CSR activities for stakeholders.

Finally, trust was shown to have a positive link with brand loyalty. Customers with high levels of trustfulness and transparency regarding a restaurant tend to show a higher level of brand loyalty, as noted by previous research (Bowden, 2009). The results demonstrate customer brand loyalty formation in the context of a halal ethnic restaurant franchise. CSR activities lead to higher brand loyalty among customers. This study proposed a process of brand loyalty among customers in the context of a halal ethnic restaurant franchise. The proposed model consists of multiple dimensions of CSR activities and suggests a mediating effect of trust between the CSR dimensions, supportive communication intention, and brand loyalty. The results enrich previous literature on the restaurant industry and demonstrate the importance of using CSR initiatives for core stakeholders as part of sustainable restaurant management.

### Practical implications

The proposed model can clarify the ways to enhance brand loyalty and sustain the trustful and emotive value of an ethnic restaurant franchise. The antecedents of CSR initiatives focus on core stakeholders and can lead to the formation of customer brand loyalty mediated by trust and affective commitment among customers. The results provide important implications for the planning of CSR engagement in the restaurant industry from the practical and managerial perspectives.

This study included four types of CSR activities for core stakeholders (i.e., communities, employees, environment, and customers) in the proposed model. CSR for employees was found to be the dimension most influential on trust. Previous research has indicated that ethical leadership and high moral values in organizational culture are linked to employees’ ethical behavior and relevant stakeholders (Al Halbusi et al., 2019, 2020). Restaurant owners and managers provide job opportunities and CSR training programs. When customers are served by polite and knowledgeable employees at a halal ethnic restaurant franchise, customers have considerable opportunities to understand restaurant organizational culture and CSR activities of the restaurants through the service process of restaurant employees.

Corporate social responsibility activities for communities and the environment were also identified as important dimensions affecting trust. Restaurant owners and managers should communicate their voluntary CSR activities for core stakeholders, such as communities, employees, customers, and the environment, through mass media, social media platforms, positive WOM, and campaigns to present their effort. Sharing the CSR activities of restaurants can enhance their reputation and trustful relationship building with customers. Moreover, noticeable CSR campaigns and information help customers improve their understanding of their contributions to employees, communities, and the environment. However, CSR for customers is not linked to trust. Small- and medium-sized restaurants may use limited resources and budgets for operating CSR activities. Accordingly, these restaurants may not provide observable CSR activities. In this regard, restaurant owners and managers should share their effort related to CSR activities to customers and core stakeholders. They also need to be actively involved in CSR programs and create CSR programs for developing distinctiveness of ethnic restaurant brands.

Second, trust is an important mediator that enhances brand loyalty. Previous research (Kim and Ham, 2016; Kim and Kim, 2019) highlighted that trust is a key to increase switching costs and retaining customers. The results suggest that it is important to increase trust among customers. Moreover, research managers and scholars need to understand the consumer value of CSR for various stakeholders as well as restaurant transparency, as these factors are associated with brand loyalty. Along this line, restaurant managers and owners may promote restaurant partnerships with stakeholders for CSR events and use brand logos, symbols, and slogans to increase the awareness of CSR activities and enhance customer trust toward the restaurant brand.

Finally, trust can facilitate supportive communication intention and brand loyalty. Restaurant managers and owners can suggest that customers become CSR supporters to increase the awareness of voluntary restaurant CSR activities and to improve the engagement of customers with these events. Moreover, customers can voluntarily engage in the spread of CSR information related to the restaurant brand on online and offline platforms. As technology advances, social media platforms and online presence have increased their influences on restaurant revenue. Restaurant managers and scholars should pay attention to co-creation behaviors among customers and the positive effects on the consumption of other customers.

### Limitations and future research suggestions

The study contributes to the related body of literature by suggesting important implications derived from empirical evidence; however, the study also has several limitations. First, the results cannot be generalized because of the limitation of the convenience sampling technique. This study also collected the data from online foreign communities and social network platforms to invite customers who have visited an ethnic halal restaurant franchise. Further research should replicate this proposed model and collect data from diverse groups of customers. Second, this study focused on a halal ethnic restaurant franchise and comparative findings among other restaurant types would not necessarily occur. Therefore, in future research, data should be collected from different restaurant types and the role of CSR activities and the process of brand loyalty among customers should be examined. Lastly, this study examined CSR activities after interviewing managers at a halal restaurant franchise and modified four dimensions of CSR activities based on previous literature. In future research, a pool of CSR activities could be developed to suggest CSR dimensions and improved measurement items in the cultural context of the restaurant industry. Lastly, different methodological approaches can be used for a substantial understanding of CSR practices in ethnic franchise restaurants and customer behavior.

## Data availability statement

The raw data supporting the conclusions of this article will be made available by the authors, without undue reservation.

## Ethics statement

Ethical review and approval was not required for the study on human participants in accordance with the local legislation and institutional requirements. The patients/participants provided their written informed consent to participate in this study.

## Author contributions

DT and Y-jA contribute to conceptualization, data arrangement, and writing. All authors equally contributed to writing, editing, and supervision. All authors contributed to the article and approved the submitted version.

## Conflict of interest

The authors declare that the research was conducted in the absence of any commercial or financial relationships that could be construed as a potential conflict of interest.

## Publisher’s note

All claims expressed in this article are solely those of the authors and do not necessarily represent those of their affiliated organizations, or those of the publisher, the editors and the reviewers. Any product that may be evaluated in this article, or claim that may be made by its manufacturer, is not guaranteed or endorsed by the publisher.
